# Quantitative CT Imaging of Ventral Hernias: Preliminary Validation of an Anatomical Labeling Protocol

**DOI:** 10.1371/journal.pone.0141671

**Published:** 2015-10-28

**Authors:** Zhoubing Xu, Andrew J. Asman, Rebeccah B. Baucom, Richard G. Abramson, Benjamin K. Poulose, Bennett A. Landman

**Affiliations:** 1 Electrical Engineering, Vanderbilt University, Nashville, Tennessee, United States of America; 2 Department of Surgery, Division of General Surgery, Vanderbilt University Medical Center, Nashville, Tennessee, United States of America; 3 Radiology and Radiological Sciences, Vanderbilt University, Nashville, Tennessee, United States of America; University of Pennsylvania Perelman School of Medicine, UNITED STATES

## Abstract

**Objective:**

We described and validated a quantitative anatomical labeling protocol for extracting clinically relevant quantitative parameters for ventral hernias (VH) from routine computed tomography (CT) scans. This information was then used to predict the need for mesh bridge closure during ventral hernia repair (VHR).

**Methods:**

A detailed anatomical labeling protocol was proposed to enable quantitative description of VH including shape, location, and surrounding environment (61 scans). Intra- and inter-rater reproducibilities were calculated for labeling on 18 and 10 clinically acquired CT scans, respectively. Preliminary clinical validation was performed by correlating 20 quantitative parameters derived from anatomical labeling with the requirement for mesh bridge closure at surgery (26 scans). Prediction of this clinical endpoint was compared with similar models fit on metrics from the semi-quantitative European Hernia Society Classification for Ventral Hernia (EHSCVH).

**Results:**

High labeling reproducibilities were achieved for abdominal walls (±2 mm in mean surface distance), key anatomical landmarks (±5 mm in point distance), and hernia volumes (0.8 in Cohen’s kappa). 9 out of 20 individual quantitative parameters of hernia properties were significantly different between patients who required mesh bridge closure versus those in whom fascial closure was achieved at the time of VHR (p<0.05). Regression models constructed by two to five metrics presented a prediction with 84.6% accuracy for bridge requirement with cross-validation; similar models constructed by EHSCVH variables yielded 76.9% accuracy.

**Significance:**

Reproducibility was acceptable for this first formal presentation of a quantitative image labeling protocol for VH on abdominal CT. Labeling-derived metrics presented better prediction of the need for mesh bridge closure than the EHSCVH metrics. This effort is intended as the foundation for future outcomes studies attempting to optimize choice of surgical technique across different anatomical types of VH.

## Introduction

Ventral abdominal hernia (VH) repair is one of the most commonly performed general surgery procedures worldwide. In the United States, nearly 350,000 repairs are performed annually at an estimated total direct cost of $3.2 billion [1]. Despite the frequency of VH repair, failure rates are high, with recurrence rates estimated at between 24 and 43 percent [2]. One possible reason for these suboptimal outcomes is a lack of evidence on the most appropriate surgical approach for different patients. At present, decisions on laparoscopic versus open repair, mesh type, mesh position, and method of mesh fixation are typically driven more by the surgeon’s personal preference than by objective data [3, 4].

Multiple factors impact the success of VH repair. These factors include preoperative conditions (e.g., obesity, nicotine use, previous infections), hernia characteristics, operative technique and perioperative care (e.g., perioperative antibiotics, operative time). To date, there is no standardized method for VH classification that consistently and efficiently describes hernia characteristics. The most well-known VH classification system is the European Hernia Society Classification for Ventral Hernia (EHSCVH) [5]. This manual system can be cumbersome to use, has been unevenly accepted by surgeons, and is inconsistently applied, especially for complex hernias. The EHSCVH is semi-quantitative in that VH’s are classified based on categorical locations with limited direct assessment of hernia size. The Ventral Hernia Working Group (VHWG) proposed a hernia grading system to access patients’ risk for surgical-site occurrences based on more comprehensive clinical factors of patients and wounds [6]; however this classification system and its variant [7] are also not commonly used given that the involved factors are complicated to access. We hypothesize that a quantitative imaging approach will provide a more objective, efficient, and reproducible means of describing VH, and that this approach may inform future evidence-based research to improve VH repair outcomes.

The purpose of this paper is to present a standardized method for quantitative anatomical labeling of VH using standard of care computed tomography (CT). We propose a detailed anatomical labeling protocol to capture the clinically relevant geometric properties of both VH and the abdominal wall. Then, using a test dataset of human subjects with VH, we demonstrate both intra- and inter-rater reproducibility of our labeling protocol for generating key quantitative descriptive parameters, including VH volumes and the relationship of VH to relevant anatomical landmarks. We perform preliminary statistical tests on the ability of the derived VH properties to predict a relevant clinical endpoint (requirement for mesh bridge closure during VHR), with comparisons to predictions from EHSCVH metrics. In discussion, we present the main contributions of our approach and its potential clinical impact, compare its practical efficacy with other related efforts on VH characterization [8–10], and envision the future work.

## Methods

### Ethics Statement

All clinical data was collected from the Vanderbilt electronic medical records systems under institutional review board approval. The full name of the institutional review board is Vanderbilt Human Research Protection Program. All procedures followed were in accordance with the ethical standards of the responsible committee on human experimentation (institutional and national) and with the Helsinki Declaration of 1975, as revised in 2008 (5). Written informed consent was obtained from all patients for being included in the study. An addendum was also obtained for the specific use for this study.

### Quantitative anatomical description of VH

Since our objective was to create a comprehensive anatomical description of VH using quantitative parameters derived from CT, we began by assembling a set of quantitative parameters that would be relevant for informing clinical decision-making on VH repair (Table 1). These quantitative parameters are divided into those describing location of the VH relative to key anatomical landmarks (including the xiphoid process, umbilicus, linea alba, linea semilunaris, anterior superior and inferior iliac spines, and pubic symphysis), those describing size and shape characteristics of the VH itself (including hernia volume, ratio of hernia volume to abdominal cavity volume [8], and defect area), and those describing mechanical properties (chiefly compliance) of the abdominal wall.

**Table 1:** Clinically relevant quantitative parameters for describing VH.

Category	Example quantitative parameters	Description	Significance
Location	Point distance from xiphoid process, umbilicus, linea alba, linea semilunaris, ASIS, and pubic symphysis	Relative location of VH with respect to bony landmarks and fascial boundaries	Reference for hernia classification [5]
Size/shape	Defect area	Area of abdominal wall fascial defect	Critical to selection of surgical techniques for hernia repair [9]
	Maximum dimensions	VH range on three orientations	Reference for hernia classification [5]
	V_hernia_/V_abdomen_	Ratio of volume size between hernia sac and abdominal cavity	A normalized indicator of hernia severity [8]
Mechanical	Compliance^a^	Ability of muscular tissues to yield elastically on a force	An indicator that correlates with ease of repair and recurrence rate [11]

^a^ Not currently accessible via CT imaging

### Labeling protocol

**Fig 1:**
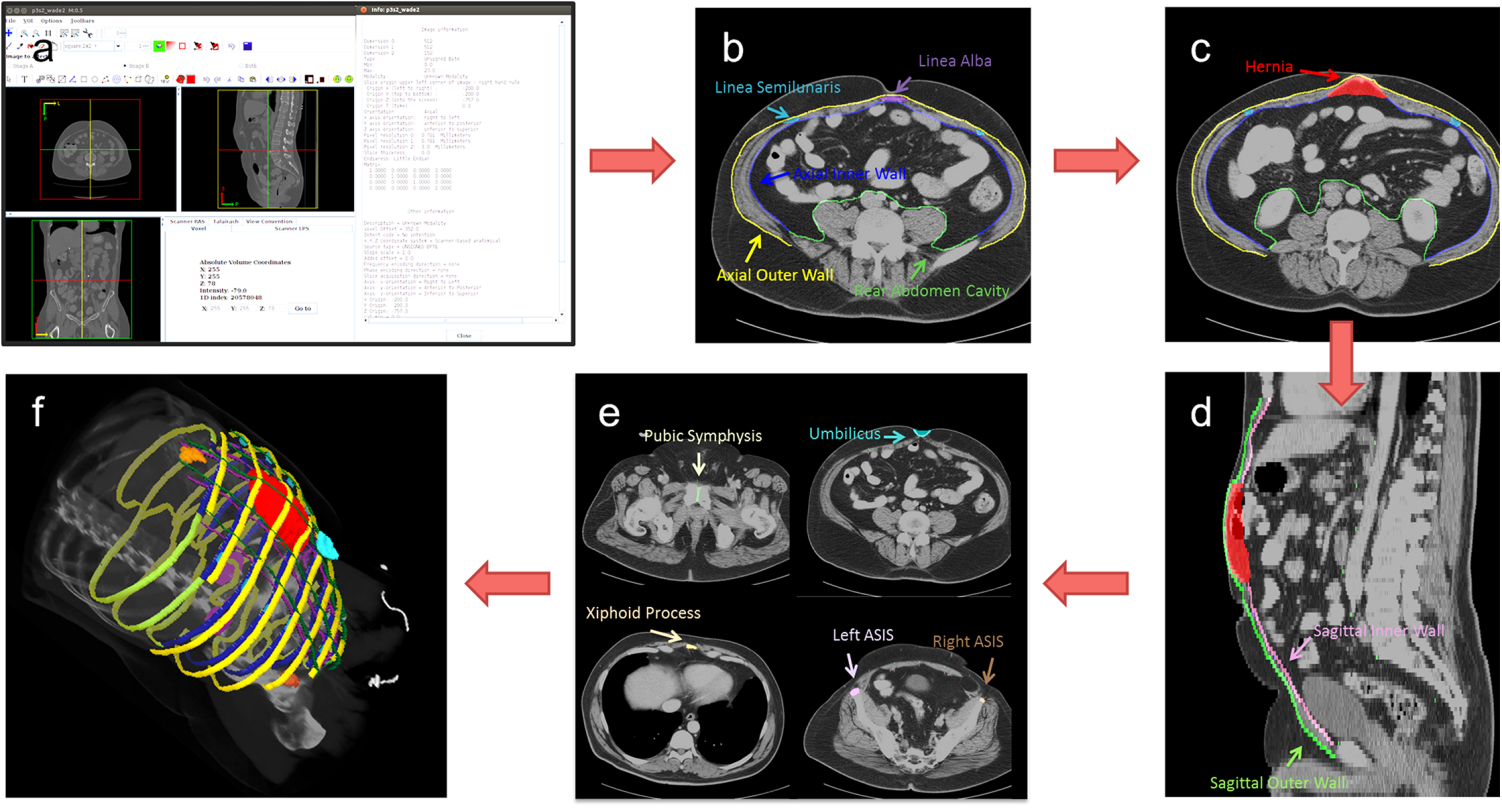
Overview of the anatomical labeling protocol. (a) Axial and sagittal slices to label are determined in terms of the size and resolution of the volume. (b) On the selected axial slices, the anterior (outer and inner borders) and posterior abdominal wall is traced. At the same time, linea alba and linea semilunaris are labeled on the appropriate axial slices. (c) The VH is labeled entirely on every axial slice where the hernia exists. (d) On the selected sagittal slices, the outer and inner borders of the anterior abdominal wall are traced. Note the previous VH and abdominal wall labels can be helpful references. (e) The umbilicus and skeletal landmarks are labeled. (f) The complete set of labels is reviewed.

**Fig 2:**
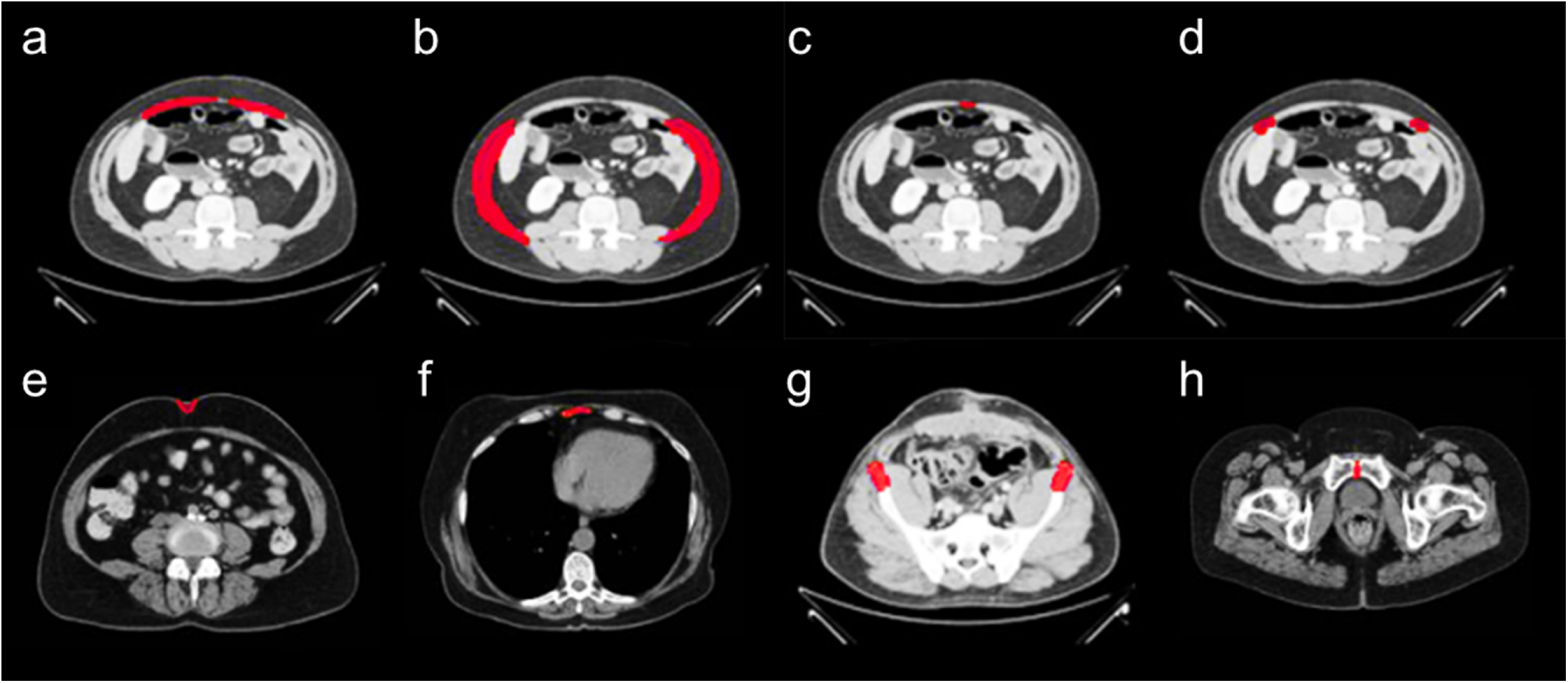
Anatomical structures included in the CT labeling protocol. (a) rectus muscles; (b) oblique abdominal muscles; (c) linea alba; (d) linea semilunaris; (e) umbilicus; (f) xiphoid process; (g) anterior superior iliac spines; and (h) pubic symphysis.

We then designed a standardized anatomical labeling protocol to enable algorithmic calculation of the above parameters (Figs 1 and 2). The protocol was created for manual implementation by a research associate with experience in anatomical labeling but without specific experience in either abdominal radiology or general surgery; the protocol is also flexible enough to be used as a foundation for future semi-automated or fully automated approaches. The detailed protocol is provided in the S1 File. Briefly, it involves the following steps: (1) Select the axial and sagittal image slices on which to label the abdominal wall. (2) Label the anterior and posterior abdominal wall, and label the linea alba and linea semilunaris on the appropriate axial slices. (3) Label the herniated region entirely. (4) Label the anterior abdominal wall on selected sagittal slices. (5) Label the skeletal landmarks and the umbilicus. (6) Review the overview of all labels. For manual labeling by a trained research associate, the entire labeling process takes approximately 1 hour for a complete abdominal CT volume.

### Data

**Fig 3:**
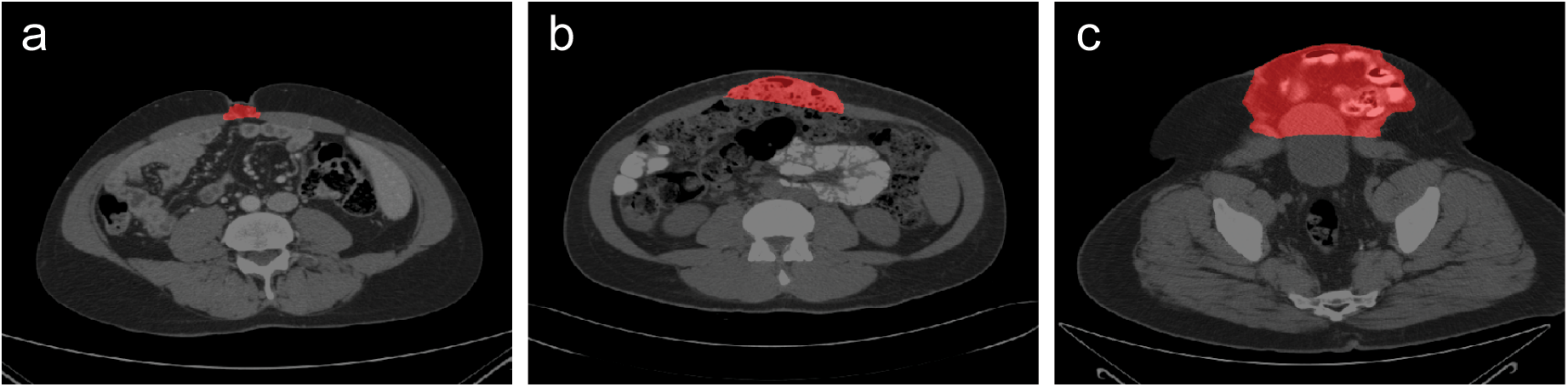
Examples of various ventral hernia sizes. (a), (b), (c) demonstrate a small, medium, and large hernia, respectively in axial slices. The herniated regions are highlighted in red.

Retrospective, clinically acquired CT data on 61 patients with suspected VHs were collected anonymously under institutional review board approval. Abdominal scans (covering from xiphoid process superiorly to pubic symphysis inferiorly) were available for the 61 patients. 18 patients were randomly selected for protocol development; an additional random 43 patients were included for a preliminary quantitative evaluation based on the protocol. Large variations were seen among the volumes in voxels (512x512x90 ~ 512x512x200) and resolution (0.6x0.6x5 mm ~ 1.0x1.0x3 mm). Average field of view in millimeters was approximately 400x400x500 mm. Various sizes of VH were observed among the involved patients (Fig 3)

### Manual labeling

A research associate was trained on the protocol using the Medical Image Processing And Visualization (MIPAV) [12] software (National Institutes of Health, Bethesda, MD) and a high resolution tablet input (Wacom, Tokyo, Japan) on a 64-bit Linux workstation. The research associate labeled all 61 datasets, with 18 of them labeled twice in randomized order with a minimum of 3 weeks between repeated volumes to ensure washout. All labels were created independently so that the research associate could not see his own prior labels. For efficiency, normal wall anatomy was evaluated on slices spaced every 5 cm. Labeling time ranged between 60 and 90 minutes per dataset. A general surgeon was also trained on the protocol and labeled a randomly selected subset of 10 of the 18 datasets. Independently, a general surgeon applied the EHSCVH protocol to the 26 patients who underwent surgical repair.

### Protocol validation

Intra- and inter-rater reliabilities were estimated from the differences between the paired results for labeling the abdominal wall, key anatomical landmarks, and the VH. For the abdominal wall, reliability was calculated using the mean surface distance (MSD) and Hausdorff distance (HD) between the two sets of labels. For key anatomical landmarks, reliability was calculated with the Euclidian distance (ED) of centroids using the centermost subcutaneous point for the umbilicus; the centroids for the xiphoid process, linea alba, and linea semilunaris; the most superior point for the pubic symphysis, and the most anterior points for the anterior superior and inferior iliac spines. For the VH, reproducibility of hernia volume was assessed by Cohen’s kappa statistic[13]. Intra-rater reliabilities were calculated on all 18 datasets. Inter-rater reliabilities were calculated on the 10 datasets for which labels were available from both the research associate and the general surgeon.

### Metrics derivation

Based on the manual labels on 61 datasets, 20 metrics were automatically derived to describe the shape, location, and surrounding environment of hernias (Table 2). Generally, the hernia shapes were directly derived from the labeled hernia volume, the hernia locations were measured from the centroids of hernia volumes to the landmarks, and the surrounding body-related metrics were calculated based on interpolated abdominal wall surfaces and segmented body masks. Thin-plate spline interpolation [14] was applied to the label meshes of the abdominal walls, which yielded an outer surface for the abdominal wall as well as a closed inner surface for the entire abdominal cavity. Fuzzy C-means clustering was used to extract the patient’s body from the scan table and background based on intensity. A further intensity clustering was applied to the extracted body region to separate fat tissue from muscles after excluding the bones and air, and thus the visceral and subcutaneous fat were discriminated by the interpolated abdominal wall surfaces [15]. Note that these 20 metrics are only a subset of quantitative parameters that we are interested in this preliminary study.

**Table 2:** Quantitative evaluations on 20 derived metrics.

Index^a^	Metric^b^	mean [min, max]
A	Hernia volume (cm^3^)	526.26 [3.99, 3946.51]
B	Hernia L-R diameter (cm)	10.69 [2.63, 30.70]
C	Hernia A-P diameter (cm)	5.11 [0.70, 17.76]
D	Hernia C-C diameter (cm)	11.64 [2.10, 30.50]
E	Hernia anterior surface area (cm^2^)	313.75 [15.28, 1269.79]
F	Hernia posterior surface area (cm^2^)	265.27 [10.14, 1103.02]
G	Average A-P hernia thickness (cm^2^)	1.16 [0.19, 3.58]
H	Normalized horizontal hernia location	0.60 [0.25, 1.34]
I	Normalized vertical hernia location	0.16 [-0.08, 0.65]
J	Distance from hernia to left ASIS (cm)	15.30 [2.24, 24.87]
K	Distance from hernia to right ASIS (cm)	16.44 [5.16, 25.12]
L	Distance from hernia to XP (cm)	23.91 [8.89, 42.92]
M	Body volume over abdomen (cm^3^)	30331.23 [19057.96, 57735.54]
N	Abdominal cavity volume (cm^3^)	8809.37 [4384.98, 18611.35]
O	Ratio of hernia to abdominal cavity volume	0.06 [0, 0.41]
P	Mean abdominal wall thickness (cm)	1.38 [0.76, 2.58]
Q	Std. of abdominal wall thickness (cm)	0.80 [0.33, 2.30]
R	Visceral fat volume (cm^3^)	2073.50 [5.94, 10322.78]
S	Subcutaneous fat volume (cm^3^)	16528.55 [296.69, 33288.25]
T	Evaluated height of abdominal region (cm)	34.46 [25.00, 40.50]

^a^ Each index represents its corresponding metric in a simpler form. A-G are considered as shape-related, G-L as location-related, and M-T as body-related metrics.

^b^ Note that (1) the quantitative values of the shape-related metrics are only collected among the subjects with identified hernias; (2) the normalized horizontal location represents the relative position from left ASIS to right ASIS, the normalized vertical location represents the relative position from the level of left and right ASIS to xiphoid process; (3) the volumetric body-related metrics are evaluated over the vertical range with labeled abdominal walls, represented as T, i.e., the evaluated height of abdominal region.

### Statistical tests

Of all 61 evaluated datasets, 26 patients underwent VH repair with intent for primary fascial closure and mesh sublay. In general, primary fascial closure is desirable during VHR whereby the hernia defect is re-approximated. When this cannot be achieved, a mesh bridge is required, leaving the original hernia defect in situ. In other words, a patient who fails to have the primary fascial closure after VHR meets the bridge requirement. These 26 patients can be classified into two groups, where 9 patients required a bridge for closure and the other 17 did not. A series of statistical tests were used to explore the clinical correlation between the derived metrics with the technical outcomes of fascial closure.

Firstly, unpaired one-tail t-test was used to examine the significant differences between the two groups for each of the 20 derived metrics.

Then, two complementary analyses based on elastic net regularized logistic regression were operated to evaluate the compound outcome prediction using multiple metrics. The goal is to construct a regression model, based on which provide an intercept *β*_0_ and a set of regression coefficients $\beta \in R^{p\times 1}$ associated with the *p* metrics to minimize the deviance of model fit to the responses given *N* observations.

$$\beta_{0},\;\beta =\;{\alpha \text{rgmin}}_{\beta_{0},\beta }\;\left(\frac{1}{N}Deviance(\beta_{0},\;\beta )+\lambda P_{\alpha }(\beta )\right)$$ (1)

where the *Deviance*(⋅,⋅) was computed under binomial distribution for logistic regression [16] to deal with binary categorical cases while estimating the odds in a continuous form. *λ* represents a non-negative regularization parameter for the penalty term *P*_*α*_(*β*).

$$P_{\alpha }\left(\beta \right)=\frac{1-\alpha }{2}{\left\|\beta \right\|}_{2}^{2}+\alpha {\left\|\beta \right\|}_{1}$$ (2)

where both the L1 and L2 norms of the regression coefficients were used for elastic net regularization [17] to constrain the regression, where some highly related metrics can be ignored (the regression coefficient approaches zero). The regularization effect is controlled by a parameter, i.e., alpha (or *α*), ranging from 0 to 1, which effectively determines the proportions of ignored regression coefficients—a larger *α* value leads to more metrics to be ignored. Note when performing the tests, the largest value was determined among a sequence of valid candidates for *λ* such that the deviance is within one standard error of the minimum, leaving *α* as the only variable.

Here, the 20 derived metrics were all included to construct the regression model that represents how multiple metrics are combined with regression coefficients to predict the technical outcomes. Specifically,

1. A predictive analysis was conducted to assess the accuracy of the predictive model using a leave-one-out cross-validation (LOOCV) scheme, i.e., building a regression model on 25 observations, and then testing it on the one left over. The continuous estimated outcomes were rounded into binary predictions for validation. By iterating this cross-validated analysis over *α* from 0 to 1 with a step size of 0.01, the numbers of remaining metrics, and those of false predictions were recorded.
2. A follow-up exploratory test was conducted by building the regression model on all 26 observations (without cross-validation) with *α* of 0.9 to identify the most predictive metrics for mesh bridge requirement after VH repair.

An additional predictive analysis using elastic net regularized logistic regression with LOOCV was conducted with the variables proposed in EHSCHV for reference. According to EHSCHV, four categorical variables, i.e., medial hernia location, lateral hernia location, recurrence of hernia, and hernia width categories, and two quantitative variables, hernia width (L-R diameter) and length (C-C diameter) acquired by clinicians were considered in place of the 20 labeling-derived metrics for regression.

## Results

### Labeling reproducibility

Of the 18 patients evaluated for labeling reliability, mean age was 50 years with 50 percent women. The mean body mass index (BMI) was 33.1 kg/m^2^. The prevalence of hernia in this population was 78%, and all of the ventral hernias were related to a previous operation (i.e. incisional hernias). The mean transverse dimension of the hernia defects was 11.4 cm.

#### Abdominal wall

Our protocol yielded high intra-rater and inter-rater reproducibility for labeling the anterior abdominal wall, with MSDs of around 2 mm and HDs of around 30 mm (Table 3). There was moderate reproducibility for the posterior abdominal wall, with an intra-rater MSD of 2.5 mm and an inter-rater MSD of 7.7 mm. The different choices on the starting and ending point of structure labeling cause the relatively large values of HD (up to 9 cm), while the MSD values are not greatly affected.

**Table 3:** Abdominal wall reliability measured by mean surfaces distances (MSD) and Hausdorff distance (HD) in mm.

**MSD**	**Axial Outer**	**Axial Inner**	**Sag. Outer**	**Sag. Inner**	**Rear Abd.**
Intra-rater	1.24±1.34	1.18±1.62	0.96±0.49	1.23±1.21	2.47±1.37
Inter-rater	1.16±0.46	1.34±0.73	1.93±1.91	2.14±1.47	7.73±2.58
**HD**	**Axial Outer**	**Axial Inner**	**Sag. Outer**	**Sag. Inner**	**Rear Abd.**
Intra-rater	19.93±17.05	16.28±16.17	19.84±13.66	20.55±22.49	36.17±24.30
Inter-rater	16.81±10.88	22.85±19.82	33.60±18.25	36.36±22.74	90.36±9.74

#### Key anatomical landmarks

Reproducibility of labels for key anatomical landmarks was moderate to high, with both intra-rater and inter-rater Euclidian distances below 5 mm (Table 4).

**Table 4:** Fascial boundaries and bony structures reliability measured by Euclidean distance (ED) of centroids in mm.

**ED**	**Xiphoid Process**	**Left ASIS**	**Right ASIS**	**Umbilicus**
Intra-rater	2.61±3.93	3.00±1.99	2.30±1.81	1.88±1.89
Inter-rater	2.43±1.84	3.51±3.15	1.83±1.39	4.69±5.91
**ED**	**Linea Alba**	**Linea Semilunaris**	**Pubic Symphysis**	
Intra-rater	4.59±5.91	4.38±3.04	1.16±1.15	
Inter-rater	3.59±2.65	4.55±1.20	2.51±1.74	

#### Hernia volumes

Reproducibility of hernia volumes was high, with an intra-rater Cohen’s kappa of 0.8 and an inter-rater Cohen’s kappa of 0.9 (Table 5).

**Table 5:** Hernia volume reliability.

Comparison	Cohen’s kappa
Intra-rater	0.83±0.05
Inter-rater	0.92±0.02

Note that we consider the proposed labeling protocol with moderate to high reproducibility with respect to the resolution (slice thickness as large as 5 mm) of the acquired CT scans.

### Quantitative evaluation

Of the 61 patients evaluated for metrics derivation, the prevalence of hernia was 72%. The mean age was 52 years with 66 percent women. The mean BMI was 33.0 kg/m^2^. The mean and range of 20 derived metrics were calculated (Table 2).

### Clinical correlation

Of the 26 patients who underwent VH repair with intent for fascial closure and were evaluated for statistical tests, the mean age was 51 years with 81 percent female with 32.1 kg/m^2^ mean BMI.

#### Unpaired one-tail t-test

Significant differences (p<0.05) were observed between two groups of patients separated by bridge closure requirement after VH repair over nine individual metrics (Table 6).

**Table 6:** Statistical comparison of 20 metrics between two groups of patients with distinct outcomes.

Index^a^	*p*-value	mean [min, max] (required bridge closure)	mean [min, max] (no bridge required)
A	0.0001^*^	823.56 [57.8, 2300.04]	107.65 [3.99, 367.81]
B	0.0000^*^	15.74 [8.51, 24.15]	7.20 [2.80, 12.80]
C	0.0029^*^	6.27 [3.05, 10.46]	3.60 [0.70, 7.24]
D	0.0004^*^	16.99 [4.50, 26.70]	8.14 [2.40, 22.20]
E	0.0000^*^	512.22 [78.93, 1036.63]	123.24 [16.30, 283.31]
F	0.0000^*^	448.13 [64.13, 1022.69]	105.59 [14.98, 271.81]
G	0.0001^*^	1.40 [0.73, 2.22]	0.72 [0.19, 1.61]
H	0.2955	0.63 [0.33, 1.01]	0.68 [0.25, 1.34]
I	0.3980	0.10 [0, 0.19]	0.09 [-0.02, 0.24]
J	0.3704	16.46 [2.24, 22.84]	15.83 [8.03, 24.87]
K	0.1834	18.54 [11.37, 25.12]	16.91 [5.16, 23.72]
L	0.4817	19.66 [13.00, 30.48]	19.55 [8.89, 30.96]
M	0.1416	32384.43 [22725.75, 42339.98]	29078.13 [19727.07, 46436.02]
N	0.4412	9233.44 [6007.36, 15065.04]	9074.89 [4683.12, 15141.70]
O	0.0005^*^	0.09 [0.01, 0.31]	0.01 [0, 0.04]
P	0.0539	1.55 [0.83, 2.44]	1.31 [0.76, 1.62]
Q	0.0083^*^	0.98 [0.39, 1.89]	0.65 [0.33, 1.07]
R	0.0655	32.48 [7.12, 65.90]	23.11 [5.94, 48.88]
S	0.1794	22960.45 [12114.26, 33288.25]	20346.47 [8218.99, 31283.60]
T	0.3847	35.68 [33.00, 38.00]	35.39 [30.30, 39.90]

^a^ Each index refers to a metric in Table 2

* indicates significant difference between the two groups

#### Predictive regression analysis

**Fig 4:**
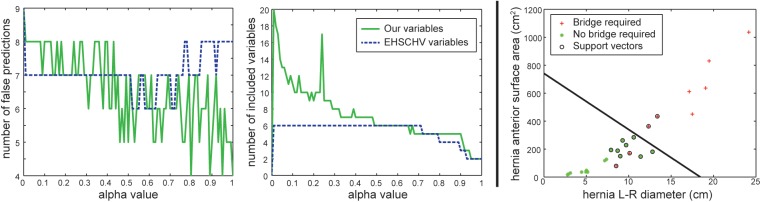
Results of preliminary statistical analyses. (a) and (b) shows the number of false predictions and number of included variables over different alpha values using cross-validated elastic net regularized logistic regression, respectively. Generally, a larger alpha value yields stronger regularization, and thus involves less variables for the regression model. Note that the blue dashed curves represent the regression results using EHSCHV variables, while the green solid curves use the variables derived from labeling. (c) presents a hyper-plane using support vector machine to separate the two groups of patients with distinct technical outcomes by the two remaining labeling-derived variables of an exploratory regression model built upon all observations.

With cross-validation, the regression model based on labeling-derived metrics and the one based on EHSCHV variables were tested. In neither case, perfect prediction was achieved. For the best case along different selection of the alpha value, the regression model using the labeling-derived metrics yields four false predictions out of 26 subjects (84.6% accuracy), where two (“hernia L-R diameter” and “hernia anterior surface area”, with *α* = 0.95 or 1.00) to five (“hernia L-R diameter”,”hernia C-C diameter”, “hernia anterior surface area”, “hernia posterior surface area”, and “average A-P hernia thickness”, with *α* = 0.80 or 0.89) metrics were used. On the other hand, the regression model using the EHS variables made at least six false predictions (76.9% accuracy), where five (except for the hernia width category) to all six variables were included (Fig 4A and 4B). With a closer look, all false predictions mentioned above were the subjects who required bridge repair but predicted as not required. These false predictions were confusing in terms of their similar hernia sizes to those having primary fascial closure after VHR.

#### Exploratory regression analysis

Only two metrics ("hernia L-R diameter" and "hernia anterior surface area") remained as the key factors for predicting mesh bridge requirement when building a logistic regression model with an alpha value of 0.9. This regression model, although used all observations, yielded the same four false estimates as the best cases using labeling-derived metrics in the predictive analysis. These two metrics can be used to identify a separating hyper-plane (a discriminative function of the two metrics) between two groups using support vector machine (SVM) [18], which represents the quantitative threshold for mesh bridge requirement. Two cases requiring bridge repair were misclassified (Fig 4C). We note that the uneven numbers between the two groups (9 vs. 17) can affect the regression model regarding its optimal threshold (we use 0.5 for logistic regression) for making predictions. Applying a SVN classifier following the regularized regression model may raise the predictive power.

## Discussion

### Main contributions

This preliminary study is intended to lay the foundation for a quantitative imaging approach to determine optimal management strategies for different subtypes of VH, and improve the surgical treatments.

First, we created a set of clinically relevant, quantitative anatomical descriptors for VH, and we designed a standardized labeling protocol to enable extraction of these parameters from routine clinical CT datasets as the foundation for future automated modeling of the relationships between VH anatomical characteristics and treatment outcomes.

Second, our protocol validation study showed acceptable inter-rater and intra-rater reproducibility for labeling the abdominal wall, key anatomical landmarks, and the VH itself. In terms of anatomical labeling, we found that abdominal wall surfaces could be appreciated on either axial or sagittal views without extensive three-dimensional visualization, while the hernia volume required tri-planar manipulation. We also found that abdominal wall surfaces are sufficiently smooth such that, for efficiency, normal wall anatomy can be labeled every 5 cm on sparse, evenly spaced slices; the entire surfaces can then be approximated by interpolation.

Next, we derived 20 quantitative parameters to describe the shape, location, and surrounding environment of VH from the anatomical labeling automatically. The collection of these metrics provide more comprehensive characteristic of VH than the available clinical measurements using EHSCHV system.

Lastly, we showed the clinical correlation between the derived quantitative parameters and the technical outcomes of primary fascial closure after VH repair with preliminary statistical tests. 9 individual metrics were shown to be significantly different between patients required bridge closure and those who did not. Through predictive analyses, we presented a regression model using multiple metrics that were capable of identifying all patients who did not require bridge closure (17 out of 17), and over half of the patients who required (5 out of 9). We also found our labeling-derived metrics more predictive than EHSCHV variables for this technical outcome. We note this is the first work to correlate VH quantification into clinically meaningful disease processes.

### Potential clinical impact

Clinically, the requirement of bridging remains uncertain pre-operatively. Surgeons may give a rough prediction by eyeballing the hernia on pre-operative imaging, i.e., a larger hernia is more likely to require a mesh bridge repair. Subjective predictions can be inaccurate. Because of this uncertainty, some techniques like myofascial release are used to avoid bridging, but may cause other problems. For example, data from our institution suggests that the extra dissection of a myofascial release significantly increases the risk of surgical site infection post-operatively[19]. Thus, objective criteria for accurately predicting the bridge requirement can be clinically relevant, and change the clinical procedure significantly. For pre-operative planning, surgeons can provide objective quantities rather than subjective size description when counseling patients and planning operations. The patient and surgeon could have an estimated probability of the need for bridge closure, and this option could be weighed while taking into account other patient factors which increase the risk of postoperative infectious complications. This could also result in lifestyle modifications prior to embarking upon a surgical repair in order to decrease the morbidity of the procedure. In some instances, the knowledge that a bridge will likely be required in a patient who has significant risk of infectious complications might lead to surgeon to accept a bridge configuration and counsel the patient accordingly.

Our labeling protocol yields reasonable objective criteria for the predication of bridge requirement. Predictive (cross-validated) statistical analysis indicates that one needs two to four metrics to yield the best prediction of the bridge requirement. This suggests that multiple metrics should be considered together for prediction. In explanatory analysis (not cross-validated), only two variables ("hernia L-R diameter" and " hernia anterior surface area ") are needed to identify a separating hyper-plane (a discriminative function of the two variables) between the groups, which represents the quantitative threshold for the requirement of bridge repair. We note that a hernia with large L-R diameter but small anterior surface area may not necessarily require a bridge requirement, which is difficult to judge visually from 3-D CT. Therefore, these metrics are helpful as objective criteria for bridge requirement prediction. We also note that while “hernia L-R diameter” has been commonly considered as an important factor, “hernia anterior surface area” has never been focused on for VH characterization. Our labeling protocol provides the access to these parameters potentially critical to correlate specific technical outcomes. On the other hand, we find the current metrics fail to discriminate some small hernias in need of bridge repair. This is partially due to the insufficient (26) and unbalanced datasets (17 vs. 9) available in the experiment. Inclusion of more datasets could help augment the prediction of primary fascial closure. Many other factors, in addition to hernia characteristics, influence the decision to perform bridge repair. The intended goals of the operation (definitive versus staged repair), level of contamination, amount of tension on the fascia when closed, need for tissue coverage, and surgeon training can contribute to the decision-making.

### Comparison to other related efforts

Our approach takes advantage of the fact that most VH patients undergo pre-surgical CT scanning to evaluate their abdomen. At present, however, no well accepted method of VH classification exists for routine use, and therefore information from imaging is used qualitatively and subjectively to make clinical decisions based on little empirical data. The EHSCVH is the only potential classification system that has been presented as a means to classify all ventral hernias. The EHSCVH system codes for (1) categorical assignment based on the location of the hernia and cause of hernia (primary or incisional), (2) categorical assignment of hernia size (small, medium, large), and (3) linear measures of hernia size (length, width). The actual implementation of this classification system can be cumbersome, however, and although the EHSCVH was published several years ago, it has not gained widespread acceptance among surgeons. The time required of a surgeon in a busy clinical practice to determine the classification for a particular patient’s hernia often prevents its use. Additionally, there is no incentive in place for the surgeon to spend the time determining the classification since the classification scheme has not been linked to specific patient outcomes or advantageous operative techniques. Finally, there are inconsistencies between surgeons when classifying complex hernias using this system. Our predictive regression analysis showed that the EHSCVH variables were not adequate to predictive the bridge closure requirement after VH repair (Fig 4A and 4B) despite its simplicity.

**Fig 5:**
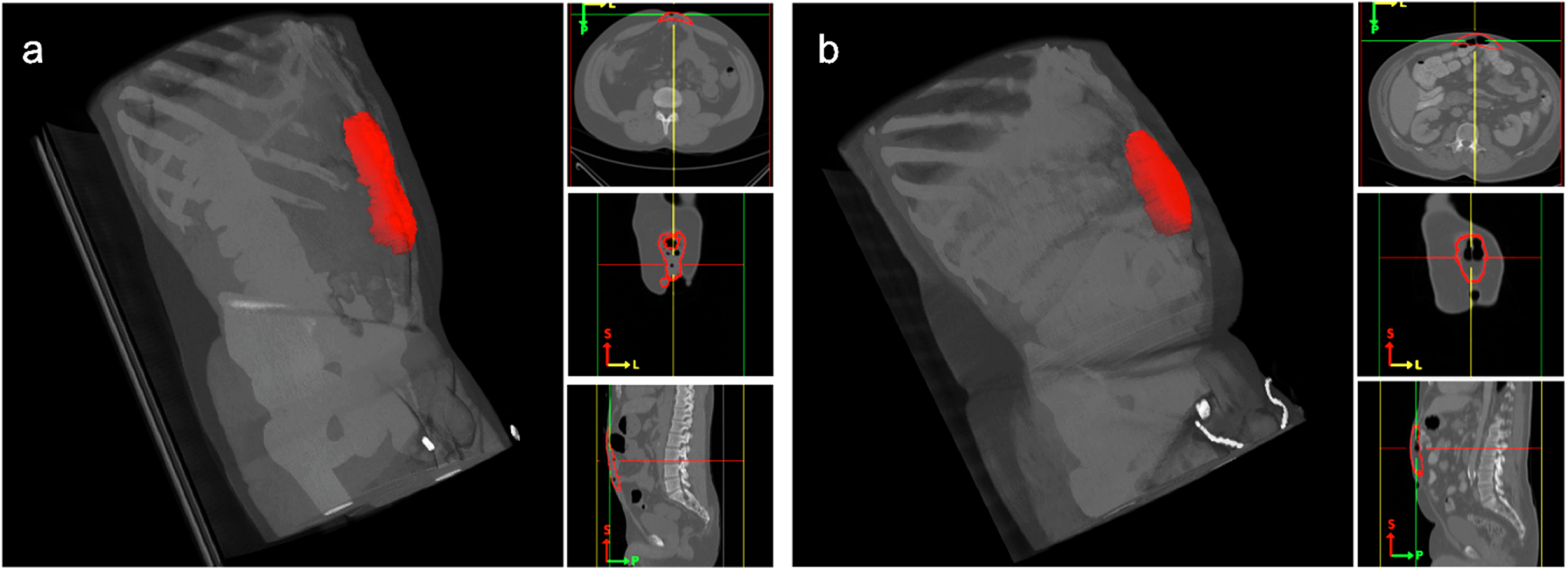
Two VH cases in volume rendering and tri-planar views. Although the two examples have almost the same hernia volume size (a = 125 cm^3^, b = 109 cm^3^), (a) is a long, shallow rupture at the umbilicus, while (b) is a short, deep protrusion of the abdominal wall. In addition, the patients’ body sizes are quite different, and the hernia in (b) is further away from the umbilicus.

A quantitative approach is attractive for two reasons: (1) it may be implemented by a trained associate or, in the future, by a semi-automated or fully automated computer algorithm, thus offloading the time for VH classification from the surgeon; and (2) its standardized nature provides a foundation for rigorous statistical correlation against patient outcomes, both retrospectively using large clinical databases and prospectively in clinical trials. At present, however, quantitative description of ventral hernias is rather rudimentary, with transverse size being the most commonly used metric. Transverse hernia size captures very little of the actual heterogeneity of VHs. Hernia volume is also inadequate for describing VH, as two VHs of the same volume may have very different shapes and may require different surgical techniques (Fig 5).

For these reasons, and for its obvious clinical implications, characterization of VH has been of interest to the image processing community in recent years. Tanaka et al. derived the volumes of the hernia sac and abdominal cavity by assuming them as ellipsoid structures with the measurement of the cranio-caudal, latero-lateral, anterior-posterior radial distances [10]. Sabbagh et al. determined the intraperitoneal volumes (including both VH and abdominal cavity) after the volume boundaries were defined using a blind-side method by a surgeon and a radiologist [8]. Yao et al. marked the required range on 3-D reconstructed CT, and measured the volumes with measurement-voluminal software [9].

**Fig 6:**
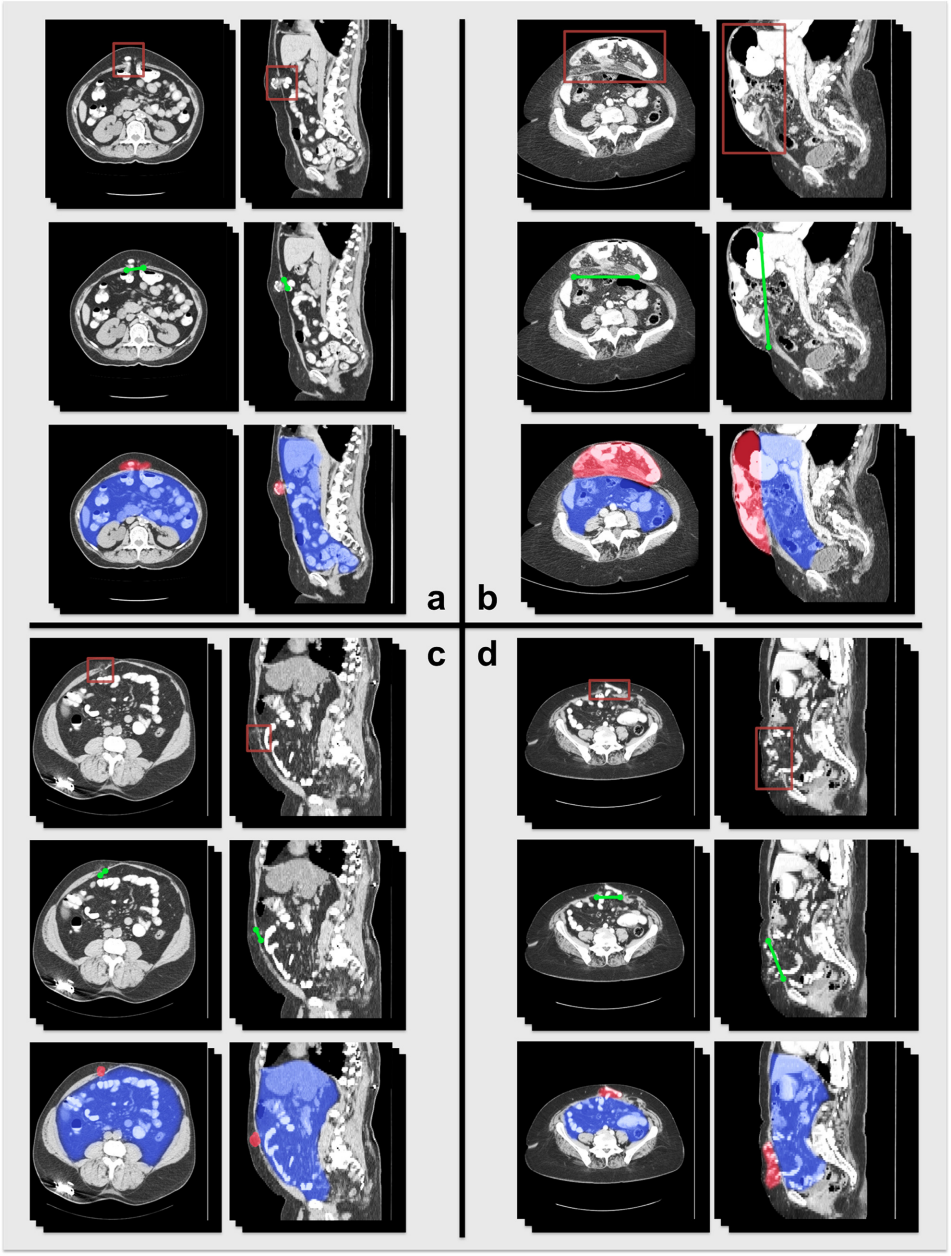
Illustration of VH characteristics on CT for four patients. In each section, the first row illustrates the location of the VH; the second row illustrates the VH defect size at the anterior abdominal wall; the third row demonstrates the volume size of the hernia sac (red) and the abdominal cavity (blue).

**Fig 7:**
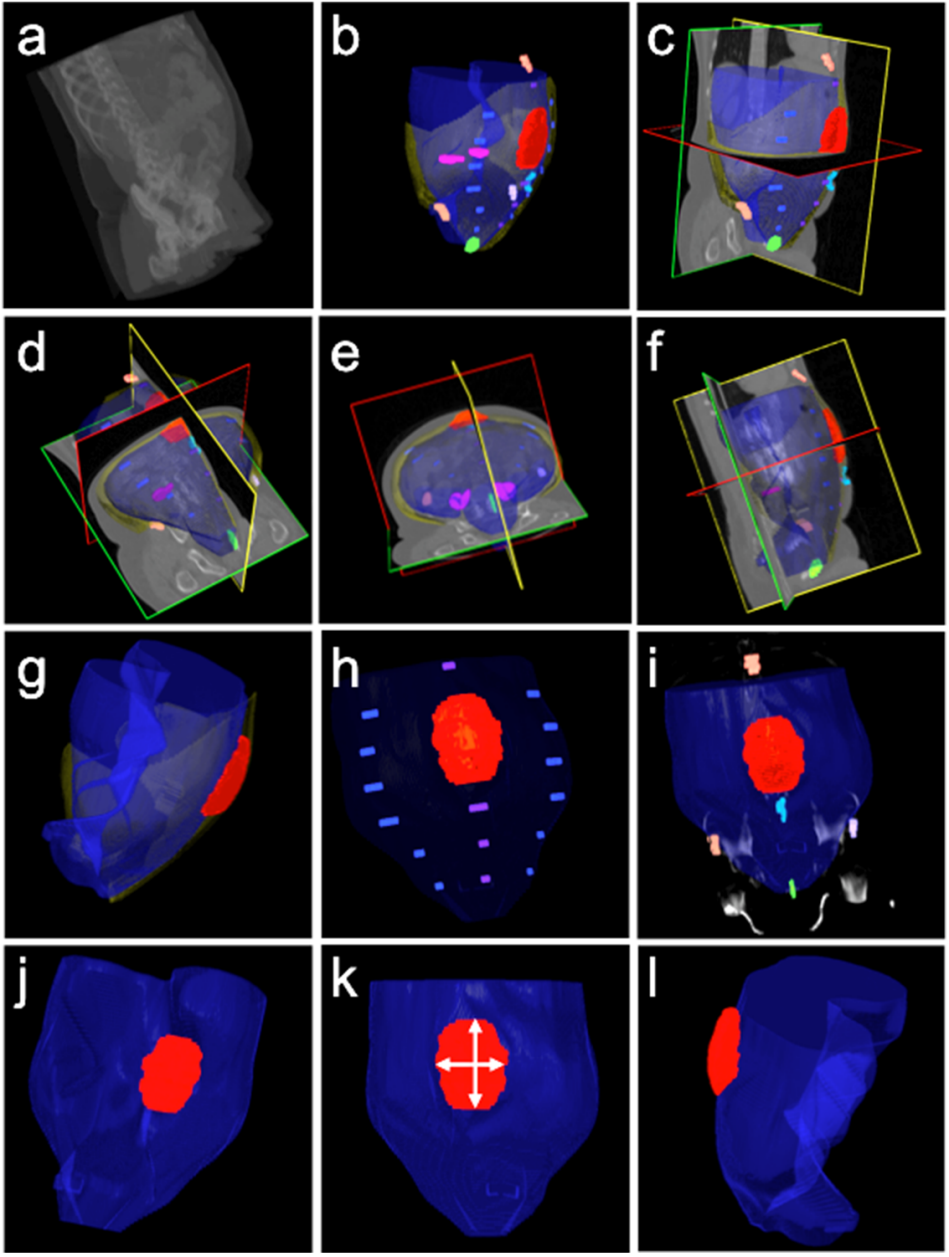
Illustration of VH characteristics in terms of processed label results. The first row, from (a) to (c), demonstrates a matchup between the original image data and the processed labels, where the abdominal walls were interpolated. The second row, from (d) to (f), demonstrates the coherence of interpolated abdominal walls with the original image in three different views. The third row, from (g) to (i), illustrates a combined model of abdominal wall and hernia volume for shape-related VH characteristics, the relative location of VH with respect to the linea alba and linea semilunaris, and the relative location of VH with respect to skeletal landmarks and the umbilicus. The fourth row, from (j) to (l), demonstrates feasibility of measuring the VH defect size, width and length of VH, and ratio of volume size between the hernia sac and the abdominal cavity.

While notable, these efforts have not addressed the fundamental challenge of measuring the complex interaction of the hernia with its biological context. Our labeling protocol allows for estimation of different geometrical properties of VH from the labeled data (Figs 6 and 7, Table 2). We consider the hernia shapes (volumetric sizes and dimensional diameters) as the chief quantitative parameters to characterize the degree of abnormality of VH. We suggest that the location of the hernia defect with respect to bony landmarks and facial boundaries is critical to VH classification. We also append metrics of surrounding structures (e.g., abdominal wall thickness, visceral and subcutaneous fat volume) as referential body status of the VH patients. These characteristics give a robust description of the hernia itself, which can then be correlated to clinical outcomes. Elastic net regularized logistic regression can be used to reduce all available variables to several key factors to predict specific technical outcomes, while sometimes a larger number of variables are required to yield better prediction. Certainly other non-hernia related factors are important in the overall definition of hernia complexity, including many clinical and patient-related factors[20], but the method described in this manuscript offers a precise and reproducible description of ventral hernia based upon specific imaging characteristics.

### Future work

One important property of VH not currently accessible via CT imaging is muscular compliance, i.e., the ability of muscular tissues to yield elastically to an applied force. This patient-specific property influences the ease of repair and may correlate with post-operative recurrence rates. Future studies may investigate the use of ultrasound or magnetic resonance elastography to estimate this property.

Also, we note that the CTs in our study were acquired at rest without performing a Valsalva maneuver. Valsalva maneuvers can be beneficial to show the potential visceral drift around the herniated region. In theory, the anatomical changes would not disrupt implementation of the proposed protocol. However, further study would be needed to explore the examine differences between scans with and without Valsalva maneuvers.

It is important to emphasize the clinical relevance of these findings. If a reproducible, quantitative, and automated method of classifying VH can be developed, the field of VH management could be significantly advanced. Namely, a reliable metric would be established whereby comparisons can be made to determine best practices—akin to cancer staging in the management of malignant disease. Relevant automated efforts are in progress[21].

## Supporting Information

S1 File Ventral Hernia Labeling Protocol. (DOCX)

## References

[1] Poulose BK, Shelton J, Phillips S, Moore D, Nealon W, Penson D, et al Epidemiology and cost of ventral hernia repair: making the case for hernia research. Hernia. 2012;16(2):179–83. Epub 2011/09/10. 10.1007/s10029-011-0879-9 . 21904861

[2] Luijendijk RW, Hop WC, van den Tol MP, de Lange DC, Braaksma MM, JN IJ, et al A comparison of suture repair with mesh repair for incisional hernia. N Engl J Med. 2000;343(6):392–8. Epub 2000/08/10. 10.1056/NEJM200008103430603 . 10933738

[3] Forbes SS, Eskicioglu C, McLeod RS, Okrainec A. Meta-analysis of randomized controlled trials comparing open and laparoscopic ventral and incisional hernia repair with mesh. Brit J Surg. 2009;96(8):851–8. 10.1002/Bjs.6668 . 19591158

[4] Millikan KW. Incisional hernia repair. Surg Clin N Am. 2003;83(5):1223–34. 10.1016/S0039-6109(03)00129-4 . 14533912

[5] Muysoms F, Miserez M, Berrevoet F, Campanelli G, Champault G, Chelala E, et al Classification of primary and incisional abdominal wall hernias. Hernia. 2009;13(4):407–14. 10.1007/s10029-009-0518-x 19495920 PMC2719726

[6] Breuing K, Butler CE, Ferzoco S, Franz M, Hultman CS, Kilbridge JF, et al Incisional ventral hernias: review of the literature and recommendations regarding the grading and technique of repair. Surgery. 2010;148(3):544–58. 10.1016/j.surg.2010.01.008 20304452

[7] Kanters AE, Krpata DM, Blatnik JA, Novitsky YM, Rosen MJ. Modified hernia grading scale to stratify surgical site occurrence after open ventral hernia repairs. Journal of the American College of Surgeons. 2012;215(6):787–93. 10.1016/j.jamcollsurg.2012.08.012 22999328

[8] Sabbagh C, Dumont F, Robert B, Badaoui R, Verhaeghe P, Regimbeau J-M. Peritoneal volume is predictive of tension-free fascia closure of large incisional hernias with loss of domain: a prospective study. Hernia. 2011;15(5):559–65. 10.1007/s10029-011-0832-y 21584816

[9] Yao S, Li J-y, Liu F-d, Pei L-j. Significance of measurements of herniary area and volume and abdominal cavity volume in the treatment of incisional hernia: Application of CT 3D reconstruction in 17 cases. Computer Aided Surgery. 2012;17(1):40–5. 10.3109/10929088.2011.636453 22145789

[10] Tanaka EY, Yoo JH, Rodrigues AJ, Utiyama EM, Birolini D, Rasslan S. A computerized tomography scan method for calculating the hernia sac and abdominal cavity volume in complex large incisional hernia with loss of domain. Hernia. 2010;14(1):63–9. 10.1007/s10029-009-0560-8 . 19756913

[11] DuBay DA, Choi W, Urbanchek MG, Wang X, Adamson B, Dennis RG, et al Incisional herniation induces decreased abdominal wall compliance via oblique muscle atrophy and fibrosis. Annals of surgery. 2007;245(1):140 17197977 10.1097/01.sla.0000251267.11012.85 PMC1867936

[12] McAuliffe M, Lalonde F, McGarry D, Gandler W, Csaky K, Trus B. Medical image processing, analysis and visualization in clinical research. Computer-Based Medical Systems, 2001 14th IEEE Symposium on; 2001.

[13] Landis JR, Koch GG. The measurement of observer agreement for categorical data. Biometrics. 1977;33(1):159–74. 843571

[14] Wahba G, Society for Industrial and Applied Mathematics Spline models for observational data. Philadelphia, Pa.: Society for Industrial and Applied Mathematics (SIAM, 3600 Market Street, Floor 6, Philadelphia, PA 19104); 1990 Available from: **http://epubs.siam.org/ebooks/siam/cbms-nsf_regional_conference_series_in_applied_mathematics/cb59**.

[15] Yao J, Sussman DL, Summers RM. Fully automated adipose tissue measurement on abdominal CT SPIE Medical Imaging; 2011: International Society for Optics and Photonics.

[16] Hosmer DW, Lemeshow S, Sturdivant RX. Introduction to the logistic regression model: Wiley Online Library; 2000.

[17] Zou H, Hastie T. Regularization and variable selection via the elastic net. Journal of the Royal Statistical Society: Series B (Statistical Methodology). 2005;67(2):301–20.

[18] Cristianini N, Shawe-Taylor J. An introduction to support vector machines and other kernel-based learning methods: Cambridge university press; 2000.

[19] Ousley J, Baucom RB, Holzman MD, Ehrenfeld JM, Sharp KW, Nealon WH, et al History of MRSA Infection Considerably Increases Risk of Surgical Site Infection in Ventral Hernia Repair. J Am Coll Surgeons. 2014;3(219):S99.

[20] Slater NJ, Montgomery A, Berrevoet F, Carbonell AM, Chang A, Franklin M, et al Criteria for definition of a complex abdominal wall hernia. Hernia. 2013:1–11. Epub 2013/10/24. 10.1007/s10029-013-1168-6 . 24150721

[21] Xu Z, Allen WM, Baucom RB, Poulose BK, Landman BA. Texture analysis improves level set segmentation of the anterior abdominal wall. Med Phys. 2013;40(12):121901 10.1118/1.4828791 24320512 PMC3838426

