# Supplementary material for: Quantitative CT Imaging of Ventral Hernias: Preliminary Validation of an Anatomical Labeling Protocol
Source: PLoS One. 2015 Oct 28;10(10):e0141671. doi: 10.1371/journal.pone.0141671 (PMC4624799; doi:10.1371/journal.pone.0141671)
Supplement: S1 File — (DOCX) [file pone.0141671.s001.docx]

**Ventral Hernia Labeling Protocol**

We find that the abdominal wall surfaces can be appreciated on either axial or sagittal views without extensive three-dimensional visualization, while the skeletal landmarks and hernia volume require tri-planar manipulation.

Abdominal Wall: Axial Labeling Protocol

- The *outer abdominal wall* (Fig. 1) is defined as a single continuous contour around the abdomen, beginning laterally at the posterior termination of the oblique muscles. It is located along the superficial border of the musculature and fascia, under the fatty and dermal layers. It surrounds all oblique muscles, tracing around the anterior-most border of the linea semilunaris, the rectus muscles, and the linea alba. Inferior aspects (Fig. 1a) are traced beginning at the anterior iliac spine on both sides, following the oblique and rectus musculature medially to the linea alba. Mid-abdominal (Fig. 1b) traces begin at the posterior termination of the oblique muscles, medially to the linea alba. Superior slice (Fig. 1c) traces begin at the point of the oblique musculature closest to the anterior-most visible rib bone, and follow the anterior wall medially to the linea alba.
- At hernia location(s) (Fig. 1d), tracings follow the outer contour of musculature, hernia sac, and fascia.
- The *inner abdominal wall* (Fig. 2) is defined as a continuous contour, tracing the innermost border of the musculature and fascia of the anterior abdominal wall. It falls directly on the anterior-most border of the viscera and follows the peritoneal lining. Like the outer wall, tracings begin at the lateral or posterior-most terminations of the oblique muscles, and run on the posterior border of the linea semilunaris, rectus muscles, and linea alba. Inferior (Fig. 2a), mid-abdominal (Fig. 2b), and superior slices (Fig. 1c) begin in the corresponding locations on the inner wall as described above.
- At hernia location(s) (Fig. 2d), tracing continues until there is clear wall deviation, or a fascial defect, due to the hernia. At that point of deviation, tracing terminates when the wall is no longer deviated.
- The *posterior abdominal cavity* (Fig. 3) is defined as a continuous contour, tracing the innermost border of the abdominal cavity as a continuation of the inner abdominal wall. It closely follows the shape of the spinal and trunk musculature and the peritoneum along the lateral borders of the abdominal cavity. The tracing follows laterally and posteriorly the majority of the viscera, only deviating anteriorly to follow the peritoneum in exclusion of the kidneys, vena cava, and aorta (retroperitoneal structures). The posterior abdominal cavity tracing must form a complete, closed shape with the inner abdominal cavity. Inferior (Fig. 3a) and medial slices (Fig. 3b) begin in the corresponding locations as described above, where the inner abdominal cavity terminates laterally and posteriorly. Superior slices (Fig. 3c) should follow the thoracic diaphragm directly inferior to the lungs.

Abdominal Wall: Sagittal Labeling Protocol

- The *outer abdominal wall* (Fig. 4) traces a continuous path along the superficial border of the musculature and fascia of the anterior abdominal wall. The tracing begins superiorly at the first bony structure (ribs or xiphoid process) and proceeds inferiorly to the interception with the anterior superior iliac spine or pelvis (Fig. 4a).
- At hernia location(s) (Fig. 4b), tracings follow the outer contour of musculature, hernia sac, and fascia.
- The *inner abdominal wall* (Fig. 5) tracing follows the innermost border of the musculature and fascia of the anterior abdominal wall using similar bounds as for the outer wall tracing, beginning superiorly at the ribs or xiphoid process and ending inferiorly at the closest point to the iliac crest or pelvic bone (Fig. 5a).
- At hernia location(s) (Fig. 5b), tracing continues until there is clear wall deviation, or a fascial defect, due to the hernia. At that point of deviation, tracing terminates when the wall is no longer deviated.

Fascial Boundary Labeling Protocol

- The *linea alba* (Fig. 6a), running directly down the midline of the abdomen, is labeled axially in order to determine its average width. The label covers the thinned area between the two rectus muscles and between the axial outer abdominal label and the axial inner abdominal wall label, only in slices where the wall is labeled.
- If hernia location(s) overlap with the linea alba, the hernia label takes precedence. The linea alba label may not exist at every slice in which the wall is labeled.
- The left and right *linea semilunaris* (Fig. 6b) are labeled axially in order to determine the average width of the fascial boundaries. The label covers the thinned area, or aponeurosis, between each rectus muscle and the obliques, and between the axial outer abdominal label and the axial inner abdominal wall label, only in slices where the wall is labeled.
- If hernia location(s) overlap with either linea semilunaris, the hernia label takes precedence. The linea semilunaris labels may not exist at every slice in which the wall is labeled.

Tri-planar, Three-dimensional Protocol

- *Hernias* (Fig. 7) are labeled volumetrically as opposed to a surface tracing. The entire hernia is fully labeled in every slice that includes the hernia. The hernia label includes all viscera that have been displaced from the abdominal cavity.
- The *xiphoid process* (Fig. 8c), the *left ASIS* and the *right ASIS* landmarks (Fig. 8d) are labeled volumetrically in at least five slices. The xiphoid process should include the five inferior-most slices that include the xiphoid process, and the two ASIS landmarks should be centered around the slices that include the anterior-most iliac point on each side.
- The entire *umbilicus* (Fig. 8b) and *pubic symphysis* (Fig. 8a) are labeled volumetrically, in all slices that include these landmarks. Note that the umbilicus includes the external indention and indication of the umbilicus, as well as the internal residual structures.


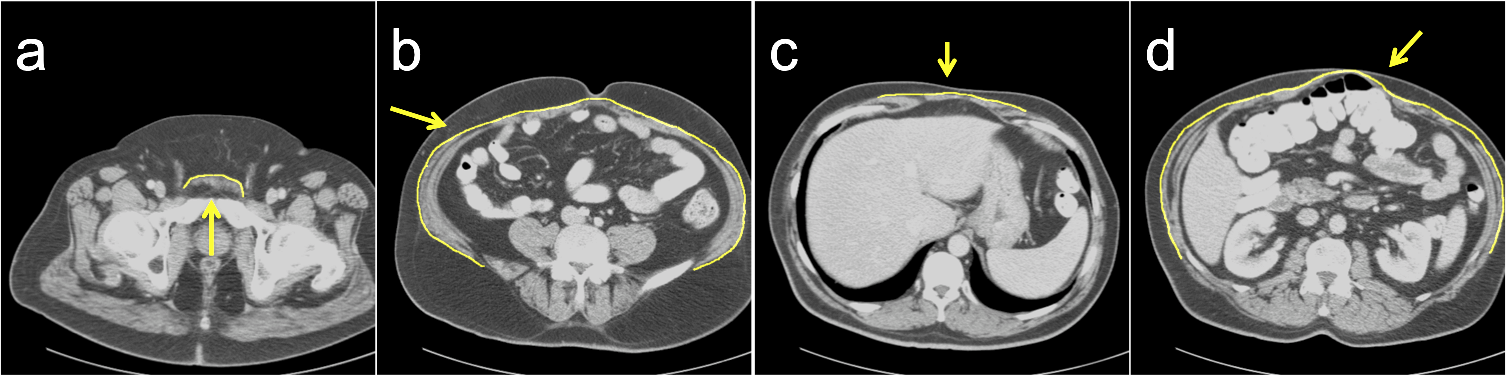


**Fig. 1** Illustration of axial labels for outer abdominal wall.


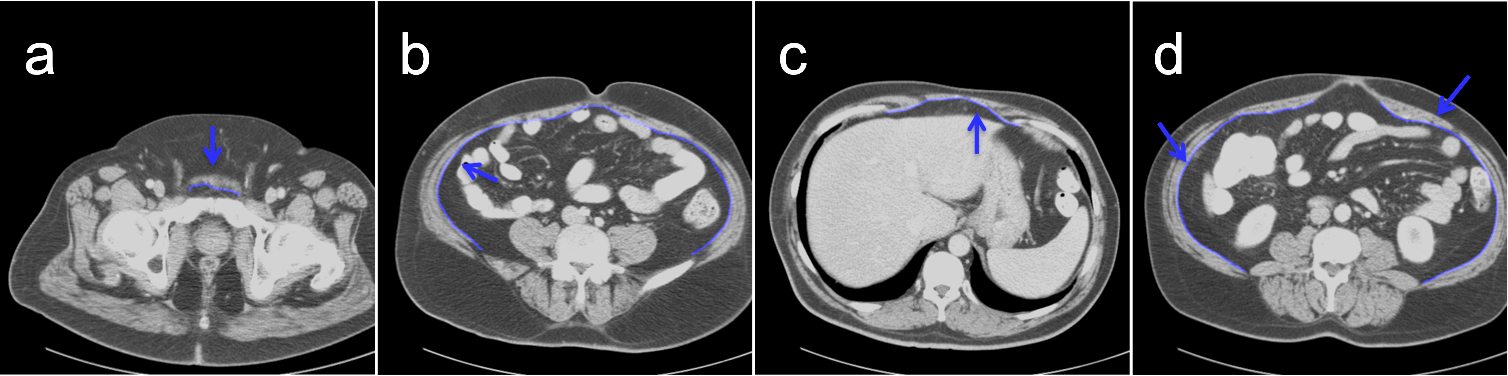


**Fig. 2** Illustration of axial labels for inner abdominal wall.


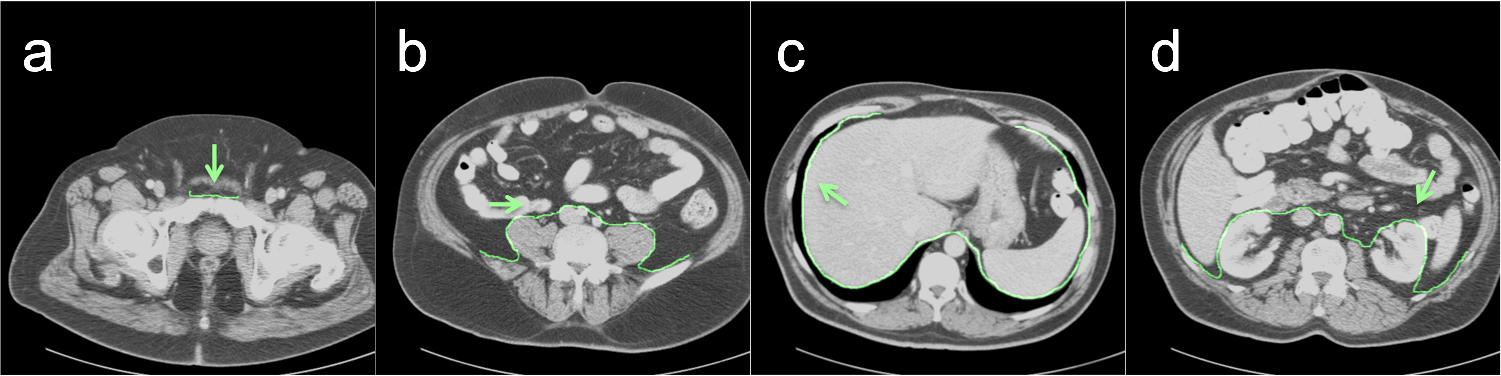


**Fig. 3** Illustration of labels for rear abdominal cavity.


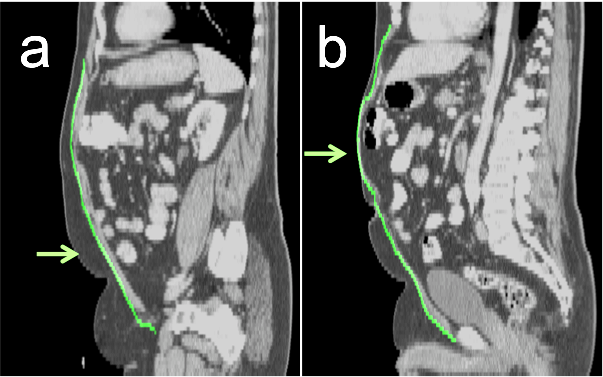


**Fig. 4** Illustration of sagittal labels for outer abdominal wall.


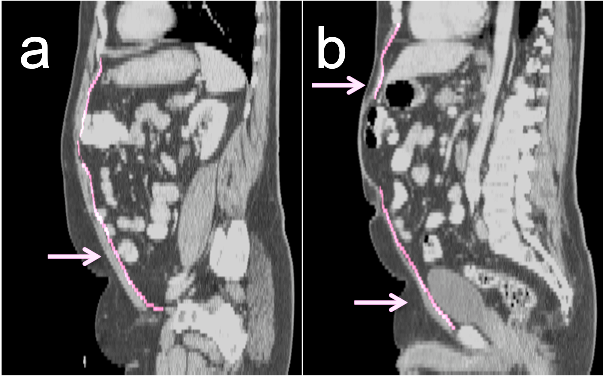


**Fig. 5** Illustration of sagittal labels for inner abdominal wall.


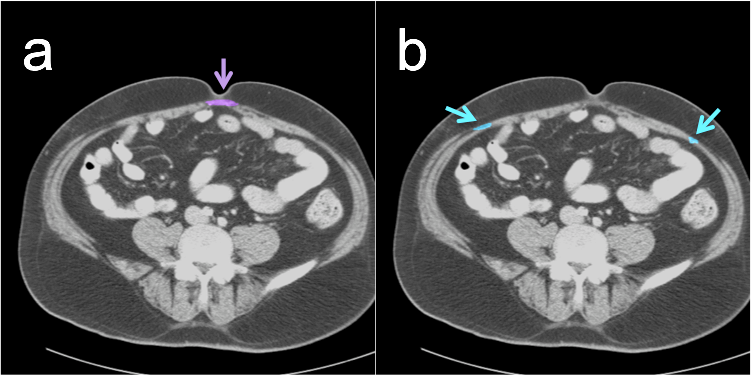


**Fig. 6** Illustration of fascial boundary labels


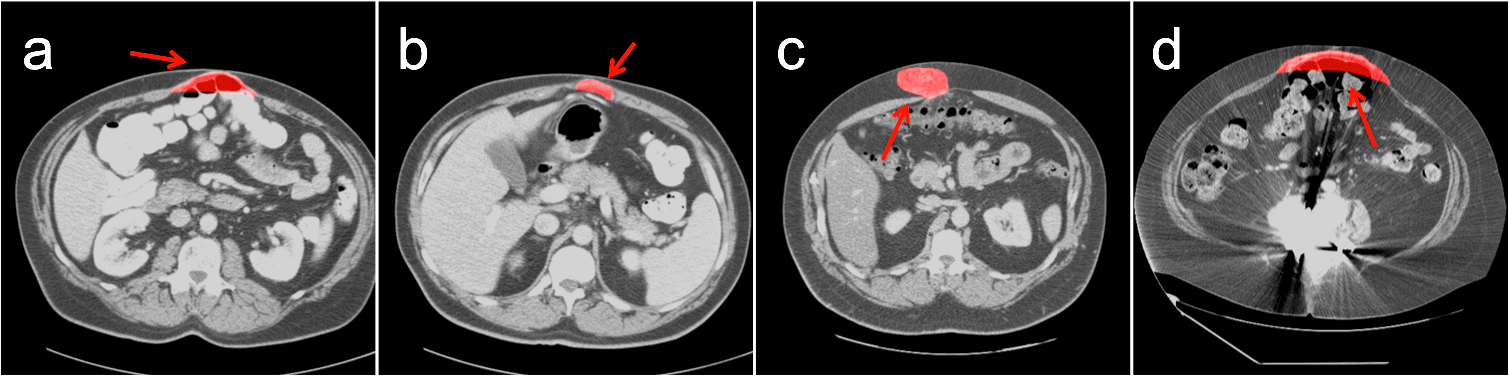


**Fig. 7** Illustration of hernia labels.


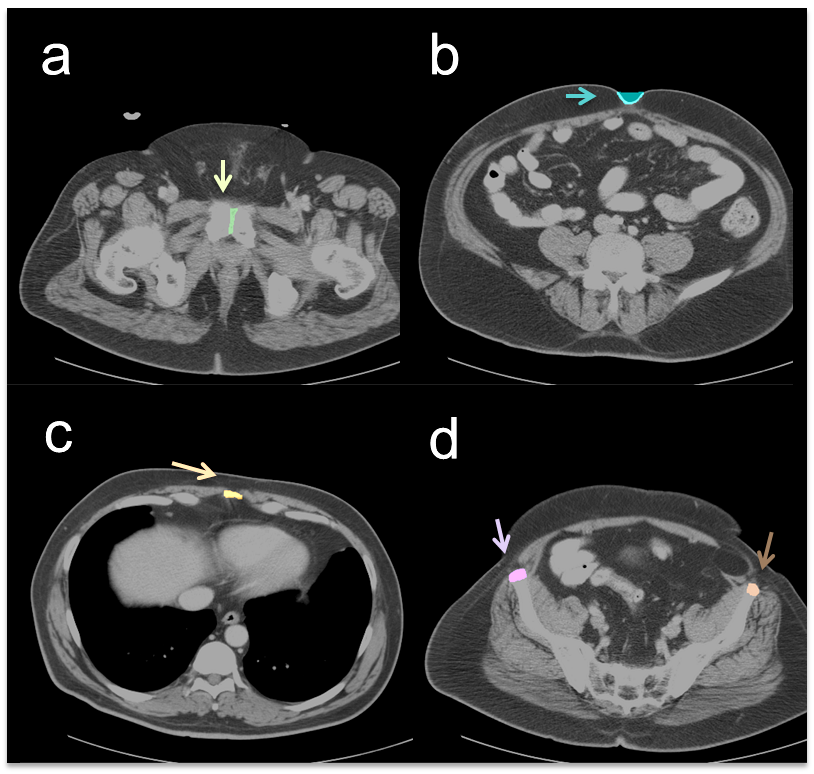


**Fig. 8** Illustration of landmark labels.
